# A teaching tool about the fickle *p* value and other statistical principles based on real-life data

**DOI:** 10.1007/s00210-020-02045-3

**Published:** 2021-01-14

**Authors:** Salem Alawbathani, Mehreen Batool, Jan Fleckhaus, Sarkawt Hamad, Floyd Hassenrück, Yanhong Hou, Xia Li, Jon Salmanton-García, Sami Ullah, Frederique Wieters, Martin C. Michel

**Affiliations:** 1 Center for Molecular Medicine Cologne (CMMC), Faculty of Medicine and University Hospital of Cologne, University of Cologne, Cologne, Germany; 2 Cologne Center for Genomics (CCG), University of Cologne, Cologne, Germany; 3 Dept. of Internal Medicine III, Center for Molecular Medicine Cologne (CMMC), Faculty of Medicine and University Hospital Cologne, University of Cologne, Cologne, Germany; 4 Inst. of Legal Medicine, Faculty of Medicine and University Hospital Cologne, University of Cologne, Cologne, Germany; 5 Inst. for Neurophysiology, Faculty of Medicine and University Hospital Cologne, University of Cologne, Medical Faculty, Cologne, Germany; 6 Biology Department, Faculty of Science, Soran University, Soran, Iraq; 7 Dept. of Internal Medicine I, Faculty of Medicine and University Hospital Cologne, University of Cologne, Cologne, Germany; 8 Dept. of Ophthalmology, Shanghai General Hospital, Shanghai JiaoTong University, Shanghai, China; 9 Dept of Ophthalmology, Faculty of Medicine and University Hospital Cologne, University of Cologne, Cologne, Germany; 10 Dept. of Pharmacology, Faculty of Medicine and University Hospital Cologne, University of Cologne, Cologne, Germany; 11 Cologne Excellence Cluster on Cellular Stress Responses in Aging-Associated Diseases (CECAD), University of Cologne, Cologne, Germany; 12 Dept. of Neurology, Faculty of Medicine and University Hospital Cologne, University of Cologne, Cologne, Germany; 13 Dept. of Pharmacology, Johannes Gutenberg University, Langenbeckstr. 1, 55131 Mainz, Germany

**Keywords:** Replicability, Statistical analysis, Teaching, P value

## Abstract

A poor understanding of statistical analysis has been proposed as a key reason for lack of replicability of many studies in experimental biomedicine. While several authors have demonstrated the fickleness of calculated *p* values based on simulations, we have experienced that such simulations are difficult to understand for many biomedical scientists and often do not lead to a sound understanding of the role of variability between random samples in statistical analysis. Therefore, we as trainees and trainers in a course of statistics for biomedical scientists have used real data from a large published study to develop a tool that allows scientists to directly experience the fickleness of *p* values. A tool based on a commonly used software package was developed that allows using random samples from real data. The tool is described and together with the underlying database is made available. The tool has been tested successfully in multiple other groups of biomedical scientists. It can also let trainees experience the impact of randomness, sample sizes and choice of specific statistical test on measured *p* values. We propose that live exercises based on real data will be more impactful in the training of biomedical scientists on statistical concepts.

**Supplementary Information:**

The online version contains supplementary material available at 10.1007/s00210-020-02045-3.

## Introduction

An alarmingly high fraction of published research in experimental biomedicine has been found not to be reproducible or replicable (Freedman et al. 2015). Other than biases at the level of study planning and conduct, data analysis, and reporting (Szafir 2018; Erdogan et al. 2020; Vollert et al. 2020), a poor understanding and inappropriate use of statistical analysis is a prevalent cause of poor reproducibility of findings in the experimental life sciences (Colquhoun 2019; Wasserstein et al. 2019; Michel et al. 2020). As highlighted very recently, inappropriate use of statistical approaches could even lead to the invalidation of issued patents (Curfman et al. 2020). Accordingly, more than 800 experts cosigned an editorial proposing to no longer rely on statistical significance and *p* values (Amrhein et al. 2019).

Firstly, a *p* value does not tell us whether a finding is true, but only what the probability is that a difference of this or a greater magnitude would have been found by chance if no difference exists between the underlying populations. Thus, even when a difference is statistically significant, it is untrue in many cases—a phenomenon predicted a long time ago (Ioannidis 2005) and later termed “false discovery rate” (Colquhoun 2014). Second, a group difference or association may have a small *p* value but the effect size is so small that it is of doubtful biological or medical relevance, for instance when the sample size is large and/or variability within the sample is low. On the other hand, a *p* value may be large but associated with an effect size that, if true, would be biologically or medically important, for instance when the sample size is low and/or variability within the sample is high. Thus, a fixed mathematical relationship exists between effect size, variability, sample size, and *p* value within any data set. Biologically important is the effect size, but the calculated *p* value is in part dependent on variability and sample size. Third, a calculated *p* value depends on the assumption that the samples being analyzed have been taken randomly from the underlying populations, i.e., biases at the level of study design and conduct have been minimized as far as possible, for instance by randomization and blinding (Macleod et al. 2015).

A fourth problem is that random sampling of data sets from the same populations causes a wide variability in observed *p*values—a phenomenon called the “fickle” *p* value (Halsey et al. 2015). The human brain is notorious for underappreciating such fickleness, i.e., the degree of variability of *p* values based on samples coming from the same populations (Bishop 2020). Extensive simulations have demonstrated how fickle a *p* value is (Halsey et al. 2015; Van Calster et al. 2018; Bishop 2020). However, it has been our experience that this concept is difficult to communicate because many biomedical scientists are not familiar with such simulations. As participants and trainers of a course of statistics for graduate students in experimental biomedicine, we have developed a tool that turns abstract simulations into a personal experience. The key idea is to use real data from a single population, which means that in theory, multiple samples from this population should differ neither in their means nor in their variability (standard deviation). The tool has meanwhile been used in additional statistics courses for biomedical graduate students in three countries (Germany, Portugal, Turkey) and consistently found to be very helpful by the participants. Therefore, we wish to share it with a wider audience.

## Methods

We have used a dataset comprised of baseline micturition frequency of 1335 patients seeking treatment for overactive bladder syndrome from a published study (Amiri et al. 2020); this dataset is made available as an Excel file (Online Supplement I). Whereas a micturition frequency of less than 8 times a day is considered normal, this patient database includes subjects with a frequency ranging from 4 to 50. As the definition of the overactive bladder syndrome is based on the presence of urgency and not on frequency (Abrams et al. 2002), it is not unexpected that some subjects in the database have a normal micturition frequency. In the overactive bladder syndrome field, a group difference of 1.5 episodes per 24 h is considered medically meaningful because meta-analysis of many clinical studies has shown that the true difference between standard of care and placebo is no more than 1.5 episodes per 24 h (Reynolds et al. 2015).

Any statistical software package can be used to perform the exercise based on the data in Online Supplement I, but we have used the Prism software (www.graphpad.com). While full use of Prism requires a commercial license, the company makes a free temporary version available for teaching courses upon request. A Prism file we use and can be used by others is provided as Online Supplement II.

**Fig. 1:**
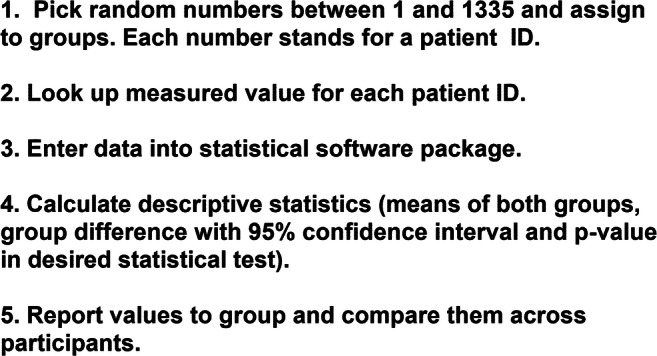
Flowchart of steps in the fickleness exercise (for details see the “Methods” section)

During the exercise, each course participant picks (preferentially random) numbers between 1 and 1335. Our example uses 40 numbers, but the exercise can be performed with any sample size. Each of these numbers corresponds to a patient ID in the Excel sheet or in the “database” data table of the Prism file (Online Supplement II). Participants then look up the measured values of the patients they have picked based on their numbers. In our example, measured values from patients 1–4 and 5–8 are then entered into the data table “*n* = 4” as groups A and B, respectively, 9–14 and 15–20 as groups A and B into the data table “*n* = 6” and 11–30 and 31–40 into the data table “*n* = 10” (those data tables are filled with dummy data in the Online Supplement II). These sample sizes were chosen because they are typical for those used in non-clinical research in biomedicine. Each of these three data tables is linked to a statistical analysis consisting of a descriptive analysis, of an unpaired *t* test and of a Mann-Whitney test (both two-tailed). The participants can easily see how outcomes differ by statistical test being applied and by sample size. They can also modify assumptions, for instance on unequal standard deviation in both groups. The participants report their results to the group and collate observed estimates of means or medians, group differences, and their 95% confidence intervals and calculated *p* values (see results). A flow-chart of tasks, particularly for those using statistical software packages other than Prism, is provided as Fig. 1. Examples based on a course with 20 participants will be presented in the “Results” section.

## Results

**Fig. 2:**
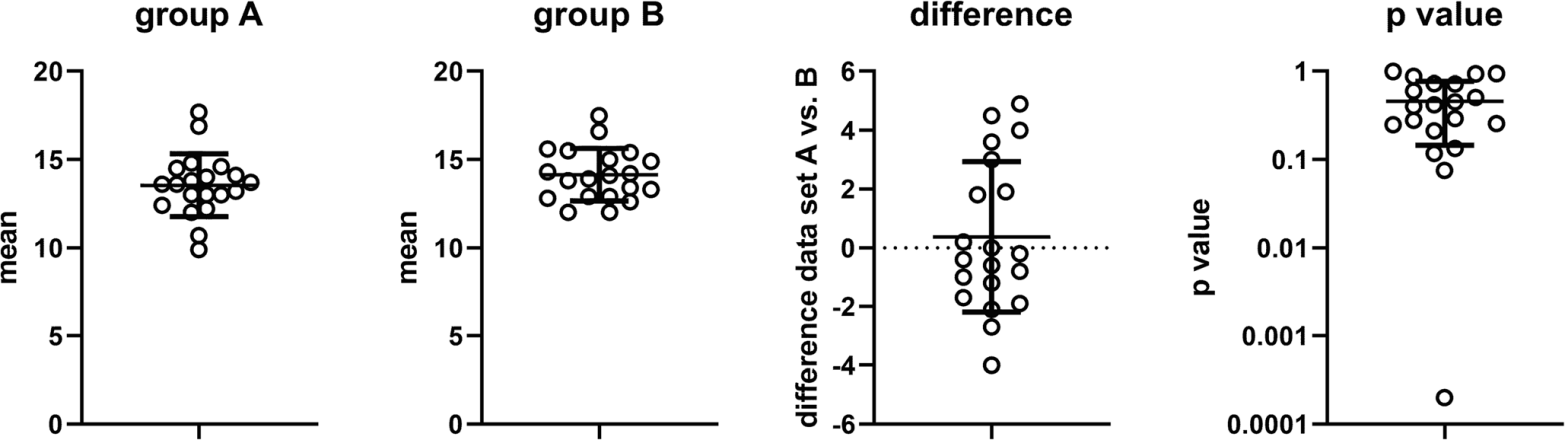
Twenty course participants had picked twice 10 random numbers and entered corresponding measured patient values as groups A and B into a Prism data table. For each sample means of groups A and B, their difference and the associated *p* value from an unpaired, two-tailed *t* test were calculated. As expected based on regression to the mean in the presence of a true null hypothesis, the mean difference was close to 0 (0.365 [95% confidence interval − 0.832; 1.562]). Each data point shows the values obtained by one participant

In our example, 20-course participants filled in the Prism sheet (Online Supplement II) for two random samples each consisting of 4, 6, or 10 subjects according to the instructions given in Methods and summarized in Fig. 1. The following is based on the data for the *n* = 10 groups; results for the *n* = 4 and *n* = 6 groups are shown in Online Supplement III. The combined group means (20 participants with 2 groups of *n* = 10) ranged from 9 to 17.7, standard deviation from 0.4 to 10.9, and the difference between groups A and B within a participant from − 4 to 4.9; associated *p* values from an unpaired, two-tailed*t* test ranged from 0.0002 to 0.9999 (Fig. 2). Corresponding values for the groups based on *n* = 4 and *n* = 6 are shown in the Online Supplement III. For comparison, the underlying population is characterized by a median of 13 (interquartile range 11; 16) and a mean ± SD of 13.65 ± 4.51 (see Online Supplement II).

## Discussion

The fickleness of *p* values has repeatedly been demonstrated based on simulations (Halsey et al. 2015; Van Calster et al. 2018; Bishop 2020). As expected by any professional statistician, *p* values vary widely between random samples drawn from the same population. As most biomedical researchers find it difficult to understand simulated data, we as trainees and trainers experienced it challenging to learn or teach about the fickleness of *p* values. Nonetheless, we and others (Colquhoun 2019; Wasserstein et al. 2019; Michel et al. 2020) feel that a sound understanding of what *p* values do and do not mean is crucial for reproducible, replicable, and robust studies and their interpretation. Therefore, we make available a large database from a real study and have developed a tool that uses them to allow scientists to experience how random choice of samples, sample sizes and choice of statistical test affect calculated *p* values. This tool was originally developed in the context of a course held at the University of Cologne but has meanwhile been tested in independent statistics courses in several other universities in Germany, Portugal, and Turkey with overwhelmingly positive feedback from the participants.

When performing experiments, we typically have little a priori knowledge about the true distribution of the variable of interest in the underlying population. We often start with a small sample (pilot experiment) and infer what the true population mean is and which variability it exhibits. What these true values are depends on the parameter of interest and the population being studied; the data being used here are just one example. However, this example illustrates based on real data how misleading a small sample can be. Ideally, more robust estimates of variability and biologically relevant effect sizes should exist before a study is done; in agreement with recent guidelines (Michel et al. 2020), we consider evidence-based power calculations important for hypothesis-testing research. As such estimates are often not feasible based on pilot experiments, we consider it good advice that research projects should be considered exploratory when meaningful sample size and power calculations are impossible due to lack of knowledge of variability and effect sizes in the underlying populations.

The example from participants of one statistics course lets participants experience how widely findings can vary between two random samples generated by the same person and between samples obtained by different people. Of note, all samples come from a single population, which means that there is no true difference, i.e., the null hypothesis is true. Experiencing this first-hand caused “wow”-effects among participants. These “wow” effects were even greater when participants learned that the group differences in number of micturitions in the random samples ranged from − 4 to 4.9 (based on the *n* = 10 examples), whereas the difference between the standard of care and placebo in the overactive bladder syndrome field (from which the samples are drawn) is less than 1.5 according to meta-analysis of micturition frequency data (Reynolds et al. 2015). Therefore, typical placebo-controlled studies in the field of overactive bladder syndrome typically include several hundred patients in each study arm (Reynolds et al. 2015). Thus, a sample size of *n* = 10 generally is accepted as being too low for the parameter for which the data were provided. However, in most cases in the experimental life sciences, we simply have no a priori knowledge on the true variability within the population for our parameter of interest. Thus, this example serves as a warning that even with *n* = 10 (representing a large sample size as compared to most experimental life science papers) does not necessarily protect from random sampling error of effect size estimates. However, the sample size is not expected to affect the distribution of *p* values under the null hypothesis. While some have argued that a minimum sample size of *n* = 5 applies to any statistical comparison of group effects (Curtis et al. 2018), we have argued against this and proposed that adequate sample sizes depend on assumptions of expected effect sizes and variability; while we agree that sample sizes of less than 5 are rarely meaningful for statistical analysis, there are examples with very large effect sizes, for instance, induction of expression of certain cytokines where smaller sample sizes are acceptable (Motulsky and Michel 2018).

Based on previous simulations (Halsey et al. 2015; Van Calster et al. 2018), our findings are entirely expected. However, the major difference is twofold: the exercise and tool are based on real data from real patients; and experiencing first-hand how different random samples can lead to different outcomes regularly surprises participants as the human brain is notorious for underestimating the variability between random samples (Bishop 2020).

The database and tool we have developed have several additional benefits: firstly, trainees can use them to “experiment” with various aspects of statistical data analysis to see how minor modifications either in statistical approach or in random sampling error affect outcomes of statistical tests; this can be done individually also by those who are not part of a formal course. Second, it allows users to experience the impact of the choice of statistical test (here: parametric unpaired t-test vs. non-parametric Mann-Whitney test). It also allows them to introduce further manipulations within the analysis options offered by Prism such as switching from tests assuming equal standard deviation to those that do not. Of note, this does not depend on the Prism software but can be applied to any statistical software package based on Online Supplement I. Third, the tool can easily be adapted if users wish for instance to work based on different sample sizes. This explicitly includes the option to introduce a “true” difference, for instance by splitting it into two databases (from sample 1–667 and 668–1335) and then adding 1 to each sample of the second group. If a true difference between groups exists (null hypothesis untrue), the distribution of *p* values will change depending on chosen sample size, which it does not if the null hypothesis is true. Fourth, as in most real-life studies and experiments, despite 1335 patients in the database only 1309 have measured values. If one of the participants coincidentally had picked a number that corresponded to a missing value, this typically sparked vivid discussions on the topic of handling missing data, another relevant aspect of generating reproducible data. Fifth, as reported in the primary publication, the clinical dataset serving as basis for the tool (Amiri et al. 2020), the underlying data deviate from a normal distribution (see graph histogram of database in Online Supplement I). This allows users to also work on other aspects such as normality testing based on real data. Sixth, using this real example can also be helpful in teaching the emphasis on reporting effect sizes with their confidence intervals rather than relying on *p* values. Finally, the database and tool are freely accessible as Online Supplements I and II of this open-access publication. We hope that this database and tool will be useful to many of our colleagues for training purposes. We explicitly encourage colleagues to modify the tool according to their needs. For instance, our course is typically run as a block of 2 days and the exercise is performed as pre-course assignment. Therefore, we encouraged participants to apply for random numbers but did not mandate that. However, if the tool is used in a course of multiple 1–2 h lessons spread over a term, it could be applied after randomization has been taught; in that setting, formal randomization could be used.

## Supplementary information

ESM 1 (XLSX 27.2 kb)

ESM 2 (PZF 851 kb)

ESM 3 (PZFX 85.9 kb)

## Data Availability

All data are made available in the “Supplementary information” section.
